# Impact of Rural Tourism Development on Residents’ Satisfaction with the Local Environment, Socio-Economy and Quality of Life in Al-Ahsa Region, Saudi Arabia

**DOI:** 10.3390/ijerph19074410

**Published:** 2022-04-06

**Authors:** Thowayeb H. Hassan, Amany E. Salem, Mostafa A. Abdelmoaty

**Affiliations:** 1 Social Studies Department, College of Arts, King Faisal University, Al Ahsa 400, Saudi Arabia; asalem@kfu.edu.sa; 2 Tourism Studies Department, Faculty of Tourism and Hotel Management, Helwan University, Cairo 12612, Egypt; 3 Independent Researcher, Giza 12573, Egypt; drsafaseefo@gmail.com

**Keywords:** rural tourism, tourism, quality of life, rural residents, Saudi Arabia

## Abstract

Tourism has a significant role in destination development, particularly in rural regions. However, within the context of the highly sensitive nature of rural areas to the ecological, economic, and socio-cultural effects of tourism development, it is important to assess the levels of satisfaction among the residents of rural destinations. The current study aimed to assess the impact of rural tourism development in the Al-Ahsa region, Saudi Arabia on the overall resident satisfaction and three relevant subdomains. The findings revealed that the three tourism development impacts under investigation, including the social, economic and environmental effects, were positively associated with resident overall satisfaction. The three influential developmental categories were also independent predictors of the satisfaction with the quality of life and environment subdomains. National policy makers are required to implement adequate rural tourism development measures and regulations to improve tourism services and activities, which would eventually be reflected in the quality of life of local residents.

## 1. Introduction

For a long period of time, tourism has been considered a significant activity that contributes directly and indirectly to the development of multiple regions worldwide. These benefits might be felt outside of urban areas, where touristic activities are a catalyst for the progress of rural and peripheral developmental plans. This is important to counteract the economic problems in the rural and agricultural environment, particularly in peripheral areas that cope with low farm income, high levels of unemployment and emigration of qualified individuals [1]. Furthermore, rural regions suffer from the lack of available options that promote the local development outside agriculture, which has led decision makers to set strategic plans for the social and environmental aspects [2,3,4]. Therefore, policy changes that target rural economies via implementing novel sectors have been conceptualized by multiple governments to overcome the negative consequences on the quality of life of rural area residents [5]. This way, the capacity of rural areas to provide accepted qualities of goods and services would be enhanced, which would reflect on the livelihoods of local residents.

Based on these findings, the ideal achievement of tourism activities in rural areas requires attaining a development in a sustainable manner. Accordingly, it is necessary to implement effective policies that enhance the development areas from a social, environmental, and economic point of view. Indeed, rural tourism (defined as traveling to rural areas for the sake of new enjoyment and experiencing the natural beauty, the quaintness of rural regions and the agricultural diversity [6]) has been a new tourism method that helps achieve the goal of rural reconstruction and urbanization by driving the economic development in multiple sectors [7]. Many rural areas are rich in promising resources that are eligible for the implementation of tourism development plans. These areas usually support the rural tourism experience, which is a central element of the tourism sector. In essence, the psychological phenomenon of rural tourism experience implies that the tourist would travel for pleasure (rather than obligation or necessity) beyond the personal life-space, and this experience would inevitably make the tourist forget the daily world while satisfying the imagined and idealized one [8,9,10]. In Saudi Arabia, there are multiple rural regions where tourists can rely on their natural resources and advantageous agritourism bases. For instance, domestic tourists can enjoy the famous landscapes and grape and pomegranate crops in Taif, the agricultural areas on the mountains of Jazan and the big olive tree farms in Al-Jouf region. Moreover, the largest date oasis in the world is in Al-Ahsa, the largest governorate in the Eastern region. Agritourism in the Al-Ahsa region can be an effective way to support community cohesion, offer job opportunities, strengthen education, achieve the sustainability goals and diversify the economic background in the region [11]. The agricultural heritage of Al-Ahsa makes it an ideal place for further touristic strategies. Within this context, many individuals prefer to leave the noise and heavy traffic behind and look for a mere natural experience in the countryside. Additionally, the numbers of domestic tourists have increased considerably because the recently spread coronavirus pandemic has postponed all plans for international touristic activities, and attention has heavily focused on strict measures in domestic gardens, parks, and recreational areas.

Based on the aforementioned observations, the development of tourism in rural areas might mediate great benefits in terms of multiple regional sectors. This can be perceived by the local residents from the economic, social and environmental aspects of tourism development. The effects of these developmental patterns on individual perceptions and satisfaction should become a matter of investigation. This is because resident satisfaction would not only reflect on their personal quality of lives, but it would augment their satisfaction with tourism development and enhance their support for future tourism development [12]. It is therefore imperative to transfer rural tourism development to fit the perceptions, values, needs and agenda of local residents to avoid the unexpected negative consequences on their quality of lives and satisfaction. In the current study, we aimed to assess the impact of three major dimensions of rural tourism development in the Al-Ahsa region, Saudi Arabia, on local resident satisfaction with their local environment, socio-economy and quality of life.

## 2. Literature Review

### 2.1. Effects of Rural Tourism Development on the Rural Environment

Tourism is generally a relatively clean industry that frequently supports the improvements of the local infrastructure, such as utilities and roads. Additionally, it helps support the protection of the environment and wildlife [13,14]. For instance, Daskin et al. [15] investigated the impact of rural tourism development in the coastal city Sinop in Turkey. Out of a five-point Likert scale score (1 to 5), the research group found the average value for positive environmental effects was 3.93 and for negative environmental effects was 2.58, which reflects the benefits of tourism to the renovation of the environment locally and the minimal negative impacts, respectively [15]. In a rural-based study in six regions in Romania [5], rural residents have perceived tourism as an important developmental factor from multiple points of view, including the natural, infrastructural and environmental spheres. The authors found an indirect link between perceived environmental effects and the support for sustainable actions, as well as another indirect link between the environmental effects and the enhancement of tourism destination [5]. However, there were some concerns regarding the awareness of local residents towards the necessity of natural conservation for effective sustained development. In addition, the residents emphasized a need for long-term planning to reduce the potential negative impacts and set appropriate protection measures [5]. Accordingly, the main goal of transforming rural regions into attractive destinations should be well-planned, considering the proper maintenance and protection of rural assets, such as the agricultural and natural resources, ecosystems and beautiful landscapes [6].

As such, in some areas, rural tourism development may be associated with negative impacts, such as environmental damage, pollution, disruption to wild life and habitat destruction [16]. In many instances, tourism development has entailed unplanned construction without adequately considering the ecological characteristics, visual consequences and environmental capacity of the destination [17]. As such, the resultant environmental damage might lead to distrust of future rural tourism development. The risk of these negative consequences can be controlled by adopting a robust set of initiatives aiming at conserving and preserving natural resources and support the sustainable development in rural areas. Nevertheless, this remains a considerable challenge [18].

### 2.2. Effects of Rural Tourism Development on the Socio-Economy

Rural areas have always been dependent on agriculture and animal husbandry. Rural tourism has the potential to revitalize the local economy, and it can bring a new direction for further developmental plans, particularly in the internet age, technological advancements, machine intelligence and construction informatization [19]. This way, policy makers can support tourism activities that attract more tourists to rural regions, enhance the infrastructure of tourist attractions, and gradually change the way by which residents rely exclusively on agriculture for livelihoods and replenish the local economy.

In the Issyk-Kul Region, Kyrgyzstan, Kozhokulov [20] showed that touristic activities have had positive impacts on the economic and social aspects in the region during the period between 2002 and 2017 despite the existence of a sharp drop of the economic benefits in 2010 due to national political instabilities. The positive impact of tourism on economy had a strong influence on the social spheres. The authors stressed the importance of tourism as a labour-intensive industry, which led to a significant reduction of unemployment rates and reducing the migration outflow among the rural population [20]. Additionally, some socioeconomic problems have been resolved via the construction of social facilities and increasing the employment of national individuals. Concomitantly, tourism preserved multiple environmental resources instead of depleting the available natural assets.

In Malaysia, rural tourism in mountainous areas has represented a rapidly growing paradigm which allows tourists to enjoy a peaceful, quiet experience while preserving the nature, the environment, and landscape in the region [21]. Furthermore, tourism has contributed to the development of the economy in the Melangkap Tiong region. Actually, many studies have underscored the importance of tourism as an important tool for preserving local cultural heritage, conserving natural resources, and replenishing the traditional activities for the sustainable development [22,23,24]. In Sinop city, Turkey, survey domains which are related to sociocultural and economic effects showed a tendency towards positive impacts [15]. However, residents were undecided with two items in the economic domain, including the rise in services and product prices and the rise in living costs. Additionally, concerning the sociocultural domain, residents were undecided about the rise in robbery rates, rise of traffic accidents, increased of illegal gambling practices, and issues related to the co-existence between tourists and local residents [15].

Recently, Zhou [19] indicated that the efficiency and competitiveness of rural tourism economy are positively correlated, and that their interaction is related to the environmental index. Interestingly, a 30% increase in the economic benefits can be attained when the competitiveness and environment are adequately coordinated [19]. However, rural regions are particularly sensitive to the potentially destabilizing effects of tourism development, especially the changes resulting from the sociocultural, ecological, and economic impacts. Therefore, sustainable development should be considered in the context of rural regional development [25].

### 2.3. Effects of Rural Tourism Development on Resident Quality of Life

As mentioned earlier, tourism development is frequently perceived as a potential driver for positive economic benefits, and this would inevitably improve the quality of life of residents. The quality of life of individuals can be studied objectively via distinct economic reflections that are external to the individuals, such as the gross domestic product (GDP) and income. Nevertheless, the objective measures might be amenable to be affected with higher GDPs (higher average income) associated with increased cost-of-living, a matter which would impact the perceived quality of life. As such, subjective assessment is critical to accurately assess true emotions, feelings of well-beings and the actual beliefs regarding the standards of livings [26,27,28]. Besides, subjective quality of life assessment would reveal the hidden drivers of tourism support [13].

In a survey-based study in Poland, Kachniewska [29] investigated the negative influences of the development of rural tourism destinations on demographic variables, housing conditions and agriculture during the period between 2009 and 2014. The results indicated that negligence and errors during early planning had led to unfavourable effects on financial, material and social costs, which was reflected on the ecology, lifestyle, and technical infrastructure. However, the influence of tourism development on the quality of life relied on internal marketing plans and proper communication during the planning stage [29]. Conversely, recent studies showed different results. In Nord-Vest in Romania, Muresan et al. [5] showed that tourism development was associated with a significant improvement in the quality of life of residents due to its impact on economic development and creating new employment opportunities. In Miaoli county in Taiwan, the majority of local residents under investigation disagreed that they had a low level of quality of life as a result of living in a touristic destination [30]. Furthermore, the economic and sociocultural benefits of tourism development in Orange county in the United States have positively supported the quality of life of residents [31].

**Figure 1:**
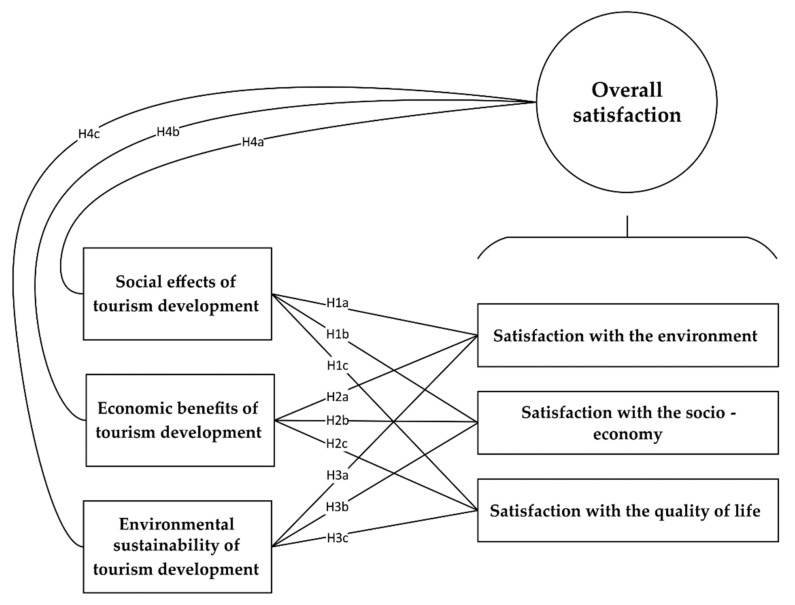
The research hypotheses of the current study.

Considering the previously mentioned facts from the literature, the authors of the current study sought to assess the impact of three variations of rural tourism development on three perceived satisfaction domains. The hypothesized model consisted of six major constructs and 12 paths of hypotheses to explore the interactive relationships between the constructs. The main research hypotheses are mentioned below, and they were summarized in Figure 1.

**H1a:**  *Perceived social effects of rural tourism development have positive impacts on resident satisfaction with their surrounding environment.*

**H1b:**  *Perceived social effects of rural tourism development have positive impacts on resident satisfaction with the socio-economy.*

**H1c:**  *Perceived social effects of rural tourism development have positive impacts on resident satisfaction with the quality of life.*

**H2a:**  *Perceived economic benefits of rural tourism development have positive impacts on resident satisfaction with their surrounding environment.*

**H2b:**  *Perceived economic benefits of rural tourism development have positive impacts on resident satisfaction with the socio-economy.*

**H2c:**  *Perceived economic benefits of rural tourism development have positive impacts on resident satisfaction with the quality of life.*

**H3a:**  *Perceived environmental sustainability practices of rural tourism development have positive impacts on resident satisfaction with their surrounding environment.*

**H3b:**  *Perceived environmental sustainability practices of rural tourism development have positive impacts on resident satisfaction with the socio-economy.*

**H3c:**  *Perceived environmental sustainability practices of rural tourism development have positive impacts on resident satisfaction with the quality of life.*

**H4a:**  *Perceived social effects of rural tourism development have positive impacts on resident overall satisfaction.*

**H4b:**  *Perceived economic benefits of rural tourism development have positive impacts on resident overall satisfaction.*

**H4c:**  *Perceived environmental sustainability practices of rural tourism development have positive impacts on resident overall satisfaction.*

## 3. Materials and Methods

### 3.1. Study Procedures and the Study Sample

A survey-based study was carried out during the period between January 2021 and February 2022. Farmers and residents of rural areas in the Al-Ahsa province were approached via an online questionnaire designated specifically for the purpose of the study. The Al-Ahsa province is in the Eastern region of Saudi Arabia with an area of about 530 km^2^, which approximately represents one-quarter of the total area of the Kingdom [32]. Although the rural population has declined from 23.4% to 17.1% during the period between 1990 and 2014 across the Kingdom of Saudi Arabia [32], rural residents represented 6.2% of the total population in the Al-Ahsa region [33]. The city is an emerging tourism destination for domestic tourists, and it is an important part of the recent efforts aiming to boast the local tourism industry and support the Saudi Vision 2030 targets [34]. It has been a part of the UNESCO Creative Cities Network since 2015 [35], and it was named “Capital of Arab Tourism” for 2019 [36]. There are 65 tourist facilities in Al-Ahsa, which are primarily located in the urban regions of Al-Hofuf and Al-Mubarraz. The province is characterized by biological diversity, natural life and wildlife in many areas [32,36]. Local destinations of touristic relevance include Al-Qara Hill, Al Ahsa National Park, and water springs. Out of the 22 attraction sites in the Eastern Province, Al-Ahsa Palm Oasis has a nature-based attraction significance. The oasis and it has become a World Heritage site in 2018 [34]. On the banks of the Palm oasis, there are several agricultural projects that attract domestic tourists.

The study survey was uploaded on Google forms and a link was created and distributed to the study participants via social media platforms. Rural residents were approached via a convenient sampling technique, where they were voluntarily provided their consent to participate. To obtain a complete record, the responses to different items were obligatory to submit a participant’s record. Therefore, a total of 274 complete records were collected via the online platform. The collected data was kept confidential, and they were used for research purposes exclusively.

### 3.2. The Study Instrument

The used questionnaire in the current study was developed based on previous validated research articles [37,38]. It comprised of three major domains (35 items), including demographic characteristics, the impacts of tourism development in rural areas and resident satisfaction. The demographic characteristics (five items) included the participant’s gender, age, marital status, level of education and the length of stay in the Al-Ahsa region. The effects of rural tourism development were categorized into three categories: the social effects (seven items), the economic benefits (six items) and the environmental impacts (two items). These items were graded on a five-point Likert scale ranging between 0 = Not effective and 4 = Very effective. The resident satisfaction domain consisted of three subdomains, including the satisfaction with local environment (seven items), satisfaction with socio-economy (five items) and satisfaction with the general quality of life (three items). Participant’s responses to the satisfaction domain were collected on a five-item Likert scale (from 0 = very dissatisfied to 4 = very satisfied).

### 3.3. Statistical Analysis

Descriptive statistics were used to express the different variables, including frequencies and percentages for categorical variables and means and standard deviations (SDs) for continuous variables. A confirmatory factors analysis was carried out to explore the convergence and discriminant validity of the constructs. The bivariate correlations between different constructs were assessed using the Pearson’s correlation test, and the outcomes were presented in a correlation matrix. A structural equation modelling (SEM) method was applied, and the following parameters were used to express the model fit: the standardized root mean square residual (SRMR), Tucker–Lewis’s index (TLI), comparative fit index (CFI), and the root mean square error of approximation (RMSEA). Finally, multivariate linear regression models were constructed to explore the independent associations between the social, economic, and environmental effects of rural tourism (each variable was entered in a separate model as a dependent variable) and each domain of participant satisfaction as an independent variable. Furthermore, the characteristics of participents were entered as potential covariates. The results of the regression analysis were expressed as beta coefficients (β) and 95% confidence intervals (95% CIs). Statistical analysis was performed using R version 4.1.1 software. A *p* value of <0.05 indicated statistical significance.

## 4. Results

### 4.1. Characteristics of the Participants

The characteristics of the respondents are demonstrated in Table 1. Approximately one-half of them were aged 21–30 years (46.4%), and most were single (50.7%). The majority of them were females (73.0%) and had a Bachelor degree (64.6%). About 44.5% of the participants had lived in Al-Ahsa for more than 15 years.

### 4.2. Confirmatory Factor Analysis

The used SEM approach utilizes a maximum likelihood (ML) method as a discrepancy method for continuous variable. This is because the ML approach usually induces symptomatic efficiency outcomes in studies with considerable sample sizes [39]. The loading values of the indicators to their constructs were checked, and the cross-loaded items were removed from the model. Consequently, three items were removed from the social effects domain, four items from the economic effects domain, and three items from the satisfaction with the environment domain.

The implemented confirmatory factor analysis approach was generally well-fitted to the data (χ^2^ = 368.98, degree of freedom [df] = 161, RMSEA = 0.069, CFI = 0.937, TLI = 0.926, SRMR = 0.047, *p* < 0.0001). As shown in Table 2, all the items were significantly loaded to their respective constructs. The internal consistency (Cronbach’s Alpha) of different domains ranged between 0.74 to 0.87, and the composite reliability ranged between 0.80 to 0.92. Furthermore, the average variance extracted (AVE) values were above the recommended values (≥0.50) [40].

To investigate the discriminant validity, the square roots of AVE were calculated and compared to the correlation coefficients as revealed by the correlation matrix. The statistical uniqueness of each domain was corroborated by the fact that the square roots of AVEs were greater than the correlation coefficients (Table 3).

### 4.3. Participant’s Responses to Different Constructs

**Figure 2:**
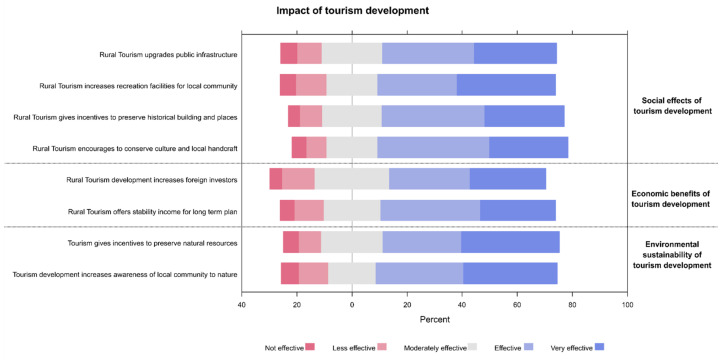
Participant’s responses regarding the effects of tourism development in the rural regions in Al-Ahsa.

The greatest effects of tourism development (effective to highly effective responses) were reported for the impact of rural tourism on culture conservation and local handcraft (68.98%) and the influence of rural tourism on preserving the historical buildings and places (66.06%). Both of those items were relevant to the social effects. Interestingly, the highest perceived economic benefits were primarily related to offering stability income for long term plan (63.50%), whereas the highest perceived effects on the environmental sustainability was focused on the role of tourism development in increasing the awareness of the local community to the nature (65.69%, Figure 2).

**Figure 3:**
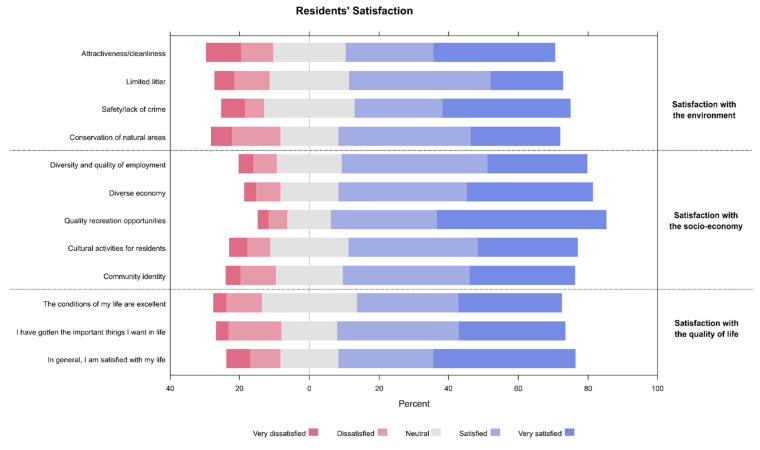
Participant’s responses regarding the different subdomains of satisfaction.

Regarding participant satisfaction, the majority of participants were satisfied with their environment (responding as satisfied or very satisfied) due to the conservation of natural areas (63.50%) and safety (61.68%). Furthermore, satisfaction with the socio-economy was heavily focused on the availability of quality recreation opportunities (78.83%) and the reliance on a diverse economy (72.99%). Interestingly, satisfaction of life was the highest perceived item in the participant’s quality of life domain (67.88%, Figure 3).

### 4.4. The Impact of Rural Tourism Development on the Overall Satisfaction

**Figure 4:**
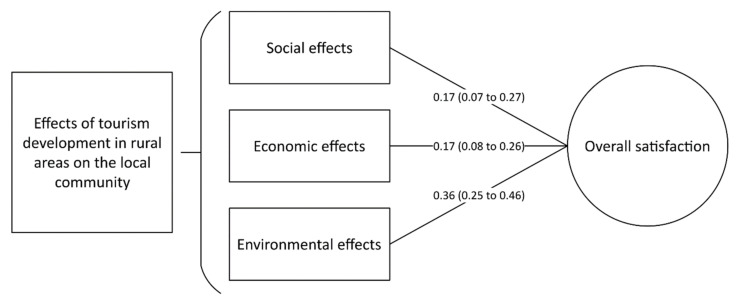
Results of the multivariate regression analysis regarding the impact of different tourism development domains on the overall satisfaction of residents in the rural regions in Al-Ahsa.

Based on the findings of the current study, the overall satisfaction with life among residents of rural regions was independently associated with the three major effects of tourism development, including social (β = 0.17, 95% CI, 0.07 to 0.27, *p* = 0.0001), economic (β = 0.17, 95% CI, 0.08 to 0.26, *p* = 0.001) and environmental effects (β = 0.36, 95% CI, 0.25 to 0.46, *p* < 0.00001, Figure 4).

### 4.5. The Impact of Rural Tourism Development on Each Construct of Participant Satisfaction

Regression models were constructed to assess the predictors of satisfaction with the environment, socio-economy, and the quality of life (Table 4). Satisfaction with the environment was positively influenced by the social (β = 0.23, 95% CI, 0.09 to 0.38, *p* = 0.002), economic (β = 0.18, 95% CI, 0.05 to 0.31, *p* = 0.007) and environmental effects of the rural tourism (β = 0.28, 95% CI, 0.12 to 0.43, *p* = 0.001). Similarly, social (β = 0.22, 95% CI, 0.08 to 0.35, *p* = 0.002), economic (β = 0.13, 95% CI, 0.01 to 0.24, *p =* 0.037), and environmental activities of rural tourism (β = 0.32, 95% CI, 0.18 to 0.46, *p* < 0.0001) were associated with satisfaction of the participants’ quality of life regardless of their demographic characteristics. However, satisfaction with the socio-economy was independently associated with two constructs of rural tourism effects, including the economic (β = 0.20, 95% CI, 0.09 to 0.31), *p* = 0.0005) and environmental effects (β = 0.47, 95% CI, 0.34 to 0.60, *p* <0.0001, Table 4).

## 5. Discussion

The main essence of rural development is to reduce the gap in regional development between urban and rural areas by augmenting the working conductions, improving the life of locals in their environment, assuring the optimal measures of quality of life, and providing privileges to rural residents that are similar to their city counterparts in a direct or indirect manner. In the present study, tourism development in the rural regions of Al-Alhsa was positively and independently associated with resident satisfaction of life. The social, economic, and environmental effects of tourism development were all apparent on the satisfaction with the environment and the resident quality of life. However, satisfaction with the socio-economy was influenced by two domains of tourism development, including the economic benefits and the environmental effects.

These results are generally consistent with those reported in the literature. For example, focusing on the social effects of tourism development, a recent study carried out in Besalú, Catalonia, Spain [41] revealed that the greatest consensus disagree was related to the negative effects on higher crime rates and the tourism-related noise, while the greatest consensus on positive statements was relevant to the fact that tourism brings jobs and preserve the cultural heritage. Similarly, although Daskin and co-authors [15] showed that residents had positive attitudes and perceptions towards the role of tourism on improving many sociocultural aspects, the authors underlined distinct negative impacts on the destination, such as the increase in travel accidents, illegal gambling and vandalism. Other studies showed that tourism in rural regions can support the participation of under-represented groups, such as the indigenous individuals and women [42,43]. Tourism development can lead also to cultural and social changes via altering power relations among the different ethnic and economic classes [44], especially in regions where development plans are limited [45]. Accordingly, the aforementioned social impacts would play an integral role in tourism development since the host community, represented as the local residents, would be in direct touch with the tourists and the positive cultural effects would help support future tourism plans [46]. Nevertheless, community-based tourism has been previously criticized for being more focused on the development of the industry itself rather than social empowerment and justice, for the failure to engage with the competitive nature of the community and for coping with the established barriers to local control [47]. Those aspects should be a matter of future research, particularly on the national level.

Interestingly, the environmental impact of tourism development had the largest influence on resident satisfaction in the current study (Figure 2). These included preserving the natural resources and promoting the awareness of local residents towards nature. Similarly, residents of Orange County in Southern California, United States, tended to agree or strongly agree that tourism supports the appearance of the local environment and help promote the restoration of natural resources and buildings [31]. On the other hand, the negative impacts of tourism were minimal for traffic congestion, pollution and solid waste problems [31]. These positive environmental effects have linearly impacted the community-related quality of life [31]. Likewise, the environmental sustainable actions of tourism development and healthy environment had a significant positive influence on the quality of life of residents in Kemiren Village, Indonesia [48]. This underlines the importance of regular monitoring of the implementation of local sustainable activities in the Al-Ahsa region to assure that no damage would take place due to excessive tourist visits. Indeed, earlier research showed that environmental protection may be perceived least of the enumerated positive effects of tourism development because the higher the number of visiting tourists the lower awareness of local residents regarding environmental preservation [49]. Therefore, the relationship between tourism and nature does not necessarily have a symbiosis of mutualism which always yields benefits. As such, these observations should be considered in future sustainable action plans.

Nevertheless, the current study has some limitations that should be discussed for future guidance of the future research. We included a relatively small number of variables in the hypothesis model under each subdomain of tourism development impacts. Future research may include more variables to assess the influence on resident satisfaction domains. Additional dependent variables might be added, and the interaction with potential moderators may be added to the hypothesized model. Another limitation is that the touristic activities might have been reduced during the study period owing the COVID-19 pandemic lockdown. Therefore, the researchers employed a convenience sampling method. Future studies might implement probability sampling methods to ensure the external validity of the study. We have also limited out study to a single province in Saudi Arabia, which might have represented an additional limitation. Large-sized and nation-wide studies are warranted to include a considerable number of rural residents in multiple spatial destinations. Finally, future study designs might benefit from the integration of rural resident perceptions, tourist responses and policymaker orientations to get reliable conclusions.

## 6. Conclusions

Rural areas have grabbed the attention of contemporary tourists worldwide. Concomitantly, these areas are vulnerable to ecological, economic, and socio-cultural effects of tourism development. The current study revealed that the social, economic, and environmental aspects of tourism development in the Al-Ahsa province, Saudi Arabia were associated with a linear increment in local resident satisfaction. Additionally, the three tourism developmental aspects were independently associated with an improved satisfaction with environment and the quality of life of individuals in the community. The socio-economic domain of satisfaction was also associated with the environmental benefits and economic aspects but not with social impacts of tourism development.

Therefore, the concept of rural tourism planning should be effectively implemented across the region. However, there are distinct gaps that need to be addressed in the Al-Ahsa province to support rural tourism development. These include the implementation of regular maintenance measures tourism-related services, ensuring the optimal levels of security and safety requirements, increasing the agricultural areas, and improving the internet services [11]. To account for the potential conflicts in the environmental-tourism relationships, local authorities in the Al-Ahsa region are encouraged to implement regulatory and quality control action plans to ensure that local residents would have high levels of awareness and active involvement in environmental protection activities. Setting up clear regulations regarding the development of agricultural and construction activities has been also cited as an important vector of rural tourism development in the region [11]. It is also important to improve tourist-related leisure services, conduct annual touristic and marketing festivals, provide convenient means of transportation, and develop the local infrastructure. These economic and environmental aspects of tourism development would not only enhance the developmental basis of the rural regions but would inevitably support the resident quality of life measures. Sustainable developmental measures must be considered to maximize the long-term economic growth rate locally and regionally.

## Figures and Tables

**Table 1:** Characteristics of the participants.

Parameter	Category	*n*	Percent
Age	21–30 years old	127	46.4%
	31–40 years old	78	28.5%
	41–50 years old	60	21.9%
	Above 61 years old	9	3.3%
Gender	Female	200	73.0%
	Male	74	27.0%
Level of Education	Primary School	2	0.7%
	Secondary School	35	12.8%
	Bachelor	177	64.6%
	Diploma	28	10.2%
	Master	22	8.0%
	PhD	10	3.6%
Marital status	Single	139	50.7%
	Married	135	49.3%
Length of Stay in Al Ahsa	<5 years	34	12.4%
	5–10 years	41	15.0%
	11–15 years	77	28.1%
	>15 years	122	44.5%

**Table 2:** The outcomes of the confirmatory factor analysis.

Constructs and Factors	SFL	AVE	CR	Cα
Social effects of tourism development		0.66	0.88	0.87
Rural Tourism upgrades public infrastructure	0.70			
Rural Tourism increases recreation facilities for local community	0.99			
Rural Tourism gives incentives to preserve historical building and places	0.77			
Rural Tourism encourages to conserve culture and local handcraft	0.75			
Economic benefits of tourism development		0.67	0.80	0.79
Rural Tourism development increases foreign investors	0.81			
Rural Tourism offers stability income for long term plan	0.83			
Environmental sustainability of tourism development		0.69	0.81	0.74
Tourism gives incentives to preserve natural resources	0.69			
Tourism development increases awareness of local community to nature	0.95			
Satisfaction with the environment		0.64	0.87	0.84
Attractiveness/cleanliness	0.91			
Limited litter	0.93			
Safety/lack of crime	0.76			
Conservation of natural areas	0.55			
Satisfaction with the socio-economy		0.65	0.90	0.87
Diversity and quality of employment	0.65			
Diverse economy	0.83			
Quality recreation opportunities	0.83			
Cultural activities for residents	0.97			
Community identity	0.70			
Satisfaction with the quality of life		0.80	0.92	0.86
The conditions of my life are excellent	0.87			
I have gotten the important things I want in life	0.90			
In general, I am satisfied with my life	0.91			

CR: Composite reliability; SFL: Standardized factor loading; AVE: Average variance extracted; Cα: Cronbach’s Alpha.

**Table 3:** A correlation matrix of the correlation between different domains.

Variables	1	2	3	4	5	6
1. Social effects	1					
2. Economic benefits	0.65 *	1				
3. Environmental effects	0.70 *	0.69 *	1			
4. Satisfaction with the environment	0.53 *	0.52 *	0.56 *	1		
5. Satisfaction with the socio-economy	0.53 *	0.59 *	0.67 *	0.63 *	1	
6. Satisfaction with the quality of life	0.55 *	0.53 *	0.59 *	0.56 *	0.61 *	1
AVE	0.66	0.67	0.69	0.64	0.65	0.80
Square root of AVE	0.81	0.82	0.83	0.80	0.81	0.89
Mean	3.79	3.75	3.73	3.67	3.77	3.98
SD	0.96	1.07	0.98	0.98	0.92	0.92

* statistically significant at *p* < 0.001.

**Table 4:** The outcomes of linear regression models to assess the independent predictors of participant’s satisfaction.

Predictor	β (95% CI) *	*t*-Value	*p*	Result
Dependent Variable: Satisfaction with the environment; Model: F(16,257) = 11.09, R2 = 0.408, Adjusted R2 = 0.372				
Social effects (H1a)	0.23 (0.09 to 0.38)	1.765	0.002	Supported
Economic benefits (H2a)	0.18 (0.05 to 0.31)	4.149	0.007	Supported
Environmental effects (H3a)	0.28 (0.12 to 0.43)	5.974	0.001	Supported
Dependent Variable: Satisfaction with the socio-economy; Model: F(16,257) = 17.1, R2 = 0.516, Adjusted R2 = 0.486				
Social effects (H1b)	0.07 (−0.06 to 0.19)	2.943	0.281	Not supported
Economic benefits (H2b)	0.20 (0.09 to 0.31)	2.9	0.0005	Supported
Environmental effects (H3b)	0.47 (0.34 to 0.60)	6.221	<0.0001	Supported
Dependent Variable: Satisfaction with the quality of life; Model: F(16,257) = 12.5, R2 = 0.438, Adjusted R2 = 0.403				
Social effects (H1c)	0.22 (0.08 to 0.35)	1.295	0.002	Supported
Economic benefits (H2c)	0.13 (0.01 to 0.24)	4.854	0.037	Supported
Environmental effects (H3c)	0.32 (0.18 to 0.46)	5.67	<0.0001	Supported

* The models were adjusted for the characteristics of participants, including the gender, age, marital status, level of education and length of stay in Al-Ahsa.

## Data Availability

Data available on request due to privacy/ethical restrictions.
